# Meningiomas with CNS invasion

**DOI:** 10.3389/fnins.2023.1189606

**Published:** 2023-06-29

**Authors:** Konstantinos Gousias, Leonidas Trakolis, Matthias Simon

**Affiliations:** ^1^Department of Neurosurgery, St. Marien Academic Hospital Lünen, KLW St. Paulus Corporation, Luenen, Germany; ^2^Medical School, Westfaelische Wilhelms University of Muenster, Muenster, Germany; ^3^Medical School, University of Nicosia, Nicosia, Cyprus; ^4^Department of Neurosurgery, Bethel Clinic, Medical School, Bielefeld University, Bielefeld, Germany

**Keywords:** invasive meningioma, CNS invasion, Simpson grade of resection, functional outcome, surgery

## Abstract

CNS invasion has been included as an independent criterion for the diagnosis of a high-grade (WHO and CNS grade 2 and 3) meningioma in the 2016 and more recently in the 2021 WHO classification. However, the prognostic role of brain invasion has recently been questioned. Also, surgical treatment for brain invasive meningiomas may pose specific challenges. We conducted a systematic review of the 2016–2022 literature on brain invasive meningiomas in Pubmed, Scopus, Web of Science and the Cochrane Library. The prognostic relevance of brain invasion as a stand-alone criterion is still unclear. Additional and larger studies using robust definitions of histological brain invasion and addressing the issue of sampling errors are clearly warranted. Although the necessity of molecular profiling in meningioma grading, prognostication and decision making in the future is obvious, specific markers for brain invasion are lacking for the time being. Advanced neuroimaging may predict CNS invasion preoperatively. The extent of resection (e.g., the Simpson grading) is an important predictor of tumor recurrence especially in higher grade meningiomas, but also – although likely to a lesser degree – in benign tumors, and therefore also in brain invasive meningiomas with and without other histological features of atypia or malignancy. Hence, surgery for brain invasive meningiomas should follow the principles of maximal but safe resections. There are some data to suggest that safety and functional outcomes in such cases may benefit from the armamentarium of surgical adjuncts commonly used for surgery of eloquent gliomas such as intraoperative monitoring, awake craniotomy, DTI tractography and further advanced intraoperative brain tumor visualization.

## Introduction

Meningiomas account for approximately 32% of the primary brain tumours (Ostrom et al., 2021). They are usually associated with a favorable prognosis after routine surgical treatments, since the vast majority are assigned to WHO ° (or CNS grade) 1 and the convexity represent their most predominant location (Sun et al., 2020; Louis et al., 2021; Ostrom et al., 2021). However, their treatment may become challenging and their prognosis more complicated in cases with deep seated lesions of the skull base, or of meningiomas infiltrating venous sinuses, or tumors with brain invasion. The surgical management of those lesions is somewhat controversial. Some consider a radical tumor removal, when safely possible, as the gold standard, while others find the Simpson grading obsolete (Sughrue et al., 2010; Gousias et al., 2016), i.e., there is no general agreement if treatment of more complex meningiomas should be guided by the concept of complete excision or cytoreduction only.

More than 60 years ago, Simpson published his classification describing the degree of meningioma resection (Simpson, 1957). According to radicality, resections are categorized in 5 groups. The oncological benefit of more radical resections was clear; better resected patients showed lower rates of recurrence. More recently, the prognostic value of the Simpson grading has been questioned (Perry et al., 1997; Sughrue et al., 2010; Chotai and Schwartz, 2022). Many surgeons decide for an incomplete resection of the tumor in order to prevent intraoperative complications and postoperative morbidity, since serial neuroimaging follow-up may allow for staged treatment aiming a tumor control rather than cure. Also, adjuvant radiotherapy and – importantly – radiosurgery may help to achieve acceptable local control rates in cases with residual tumor.

In this paper we will systematically review the recent literature on meningiomas with CNS invasion. We will specifically focus on prognostic issues. We will also investigate the relationship between extent of resection and recurrence in these tumors, as well as their surgical management and recent advances in meningioma invasion imaging.

## Methods

**Figure 1:**
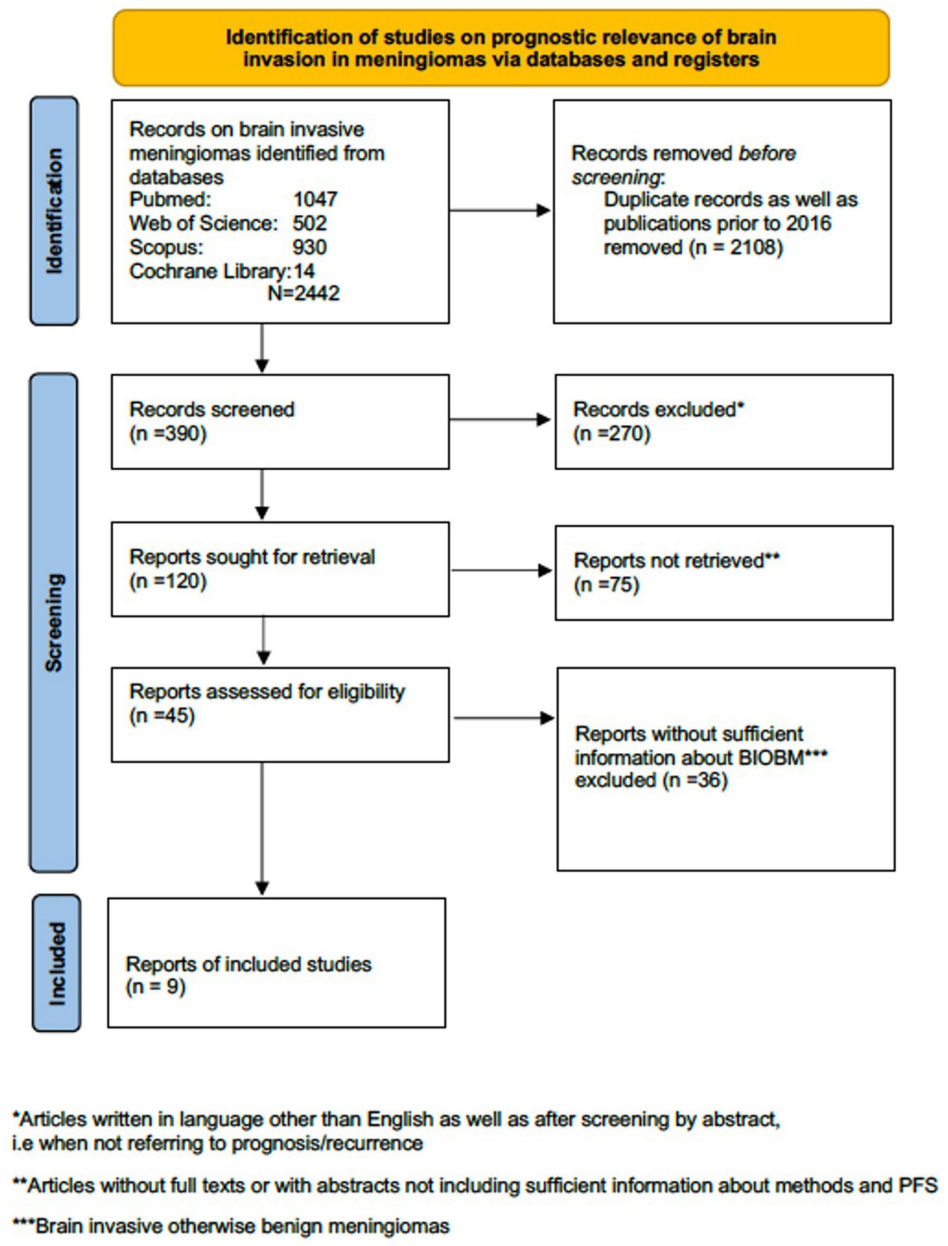
PRISMA flow diagram regarding studies on prognostic relevance of brain invasive meningioma.

We performed a systematic review of English language original articles, reviews or meta-analyses registered in the Pubmed, Scopus, Web of Science and Cochrane Library databases (1st January 2016 to 31th May 2023) according to the PRISMA guidelines using the following search terms: ‘meningioma’ and’CNS invasion’ or ‘brain invasion’ (Page et al., 2021). 2016 was chosen as the starting time in order to include only studies published after the release of the revised 4th edition of WHO brain tumor classification in 2016. No studies on the prognostic relevance of CNS invasion based on new 5th WHO edition have been identified (Figure 1). We also provide a narrative review of radiological advances and the surgical aspects of meningioma brain invasion.

## Definition and prognostic relevance of CNS invasion. A systematic review

Meningiomas that invade the skull, venous sinuses as well as the neighboring soft tissue show an aggressive clinical course and should be aggressively treated, accordingly (Gousias et al., 2016; Goldbrunner et al., 2021; Ostrom et al., 2022). High grade invasive meningiomas, in particular those with infiltration of the venous sinuses or scalp invasion, may even demonstrate, in addition to their high recurrence rate, extremely rare distant metastasis (Kessler et al., 2017; Dalle Ore et al., 2019; Bailey et al., 2023).

However, the term of invasive meningiomas refers mainly to CNS invasion.The latter has been identified as an unfavorable prognostic factor for recurrence already some decades ago (Perry et al., 1997, 1999). As a consequence, brain invasion has been included in the revised 4th edition of the WHO classification for CNS tumours in 2016 and still remains in the newest 2021 release as a stand-alone criterion for assigning a meningioma to the CNS grade 2.

It should nevertheless be noted, that brain invasive meningiomas most often demonstrate additional malignant features. Behling et al. assessed retrospectively 1718 meningiomas, 6.7% of which showed CNS invasion, and found a positive correlation between invasion and higher Ki67 proliferation rate (Behling et al., 2021). A medical history of radiation exposure may be associated with invasive growth and a higher histological grade (Goto et al., 2014; Carr et al., 2021). Radiation-induced meningiomas demonstrate higher rates of recurrence after surgery and radiotherapy, and develop in relatively younger patients at the site of previous radiation (Goto et al., 2014; Carr et al., 2021).

More recently, the prognostic relevance of a sole CNS invasion without further characteristics of atypia or malignancy (BIOBM, brain invasive but otherwise benign meningiomas) has been questioned (Baumgarten et al., 2016; Spille et al., 2016; Nakasu and Nakasu, 2021; Kim et al., 2022). Spille et al. reviewed retrospectively their institutional cohort of 467 primary meningiomas of all grades according to the 2007/2016 WHO criteria and reported a twice as high recurrence rate of brain invasive vs. noninvasive meningiomas after gross total resection. However, brain invasive but otherwise benign meningiomas WHO ° 2 showed better progression free survival (PFS), similar to benign WHO ° 1, when compared to atypical WHO ° 2 meningiomas (Spille et al., 2016). Baumgarten et al. investigated the recurrence rate in a cohort of 229 patients WHO ° 2 treated in two different brain tumor centers in Germany and also found a significant better PFS in BIOBM when compared to atypical meningiomas WHO ° 2 (Baumgarten et al., 2016). A strong limitation of the aforementioned study, though, was the short follow up (median 22 months). Kim et al. analyzed their cohort consisting of 292 meningiomas WHO ° 2 treated between 2001 and 2020, and carried out an additional meta-analysis of the available literature including 3,590 meningiomas. These authors found no consistent association between CNS invasion and PFS. However, this study did not include a central neuropathological review and the histological evaluation reported was according to the WHO criteria at the time of treatment (Kim et al., 2022). Another meta-analysis of the prognostic relevance of CNS invasion was conducted by Nakasu et al. and included studies published after 2000. CNS invasion was identified as a predictor of a shorter PFS in the combined cohort, i.e., meningiomas WHO ° 1–3, whereas BIOBM in particular showed similar recurrence rates to meningiomas WHO ° 1 (Nakasu and Nakasu, 2021). Similarly, Garcia-Segure et al. identified brain invasion as a predictor of tumor recurrence in meningioma WHO ° 2 only in cases with additional histological signs of necrosis in their cohort comprising 181 meningiomas WHO ° 2 treated between 1995 and 2015 (Garcia-Segura et al., 2020).

Studies allowing direct comparisons of BIOBM vs. remaining meningiomas WHO ° I are definitely more appropriate to analyze the prognostic relevance of sole CNS invasion. Biczok et al. investigated retrospectively a bi-institutional cohort comprising 875 meningiomas WHO ° 1 diagnosed according to the 2007 WHO criteria and treated between 2005 and 2014, and found shorter PFS in patients with BIOBM compared to the remaining population (50 vs. 68 months), which however did not reach statistical significance. Importantly, similar results were obtained in 170 patients for which tissue samples could be made available for a neuropathological review of the brain/meningioma interface. Noteworthy, brain invasion without further signs of atypia was suspiciously frequent in these specimens (16.5%) (Biczok et al., 2019). Traylor et al. reviewed a series of 543 meningiomas (339 WHO ° 1, 200 WHO °2 and 4 WHO ° 3 after neuropathological review according to WHO 2016 criteria) treated surgically in Texas Southwestern Medical Center between 1994 and 2005 and found no significant increase of recurrence risk for BIOBM vs. WHO grade ° 1. Similar to the previous study, this study includes a very high rate of WHO ° 2 (37%) and BIOBM (26.5%) (Traylor et al., 2023). Banan et al. compared the recurrence rates between 243 benign WHO ° 1 meningiomas without CNS invasion and 25 BIOBM (i.e., 9.3% of the overall cohort) treated between 2004 and 2012 and found significantly higher rates (28% vs. 4%) in BIOBM vs. remaining WHO °1 tumors. Strengths of the study design include a central neuropathological reevaluation according to the WHO criteria of 2016 as well as the use of additional immunohistochemical staining against GFAP (Banan et al., 2021). Table 1 lists all relevant meningioma studies on the prognostic relevance of CNS invasion with specific consideration of BIOBM.

**Table 1:** Prognostic relevance of invasion in brain invasive otherwise benign meningiomas WHO ° 2 (BIOBM, studies since 2016 included).

Author	No of patients with meningiomas	WHO edition	Follow-up in months	Degree of resection and recurrence*	Association of CNS invasion with recurrence
Spille et al. (2016)	401 WHO ° 1, 60 WHO ° 2 (incl. 20 BIOBM) 6 WHO ° 3	4th/rev.4th, 2007/2016 (neuropathological re-evaluation)	91	Longer PFS after GTR vs. STR, *p* = 0.025	Brain invasive vs. noninvasive meningiomas showed twice as high recurrence rates after GTR BIOBM showed better prognosis than atypical meningiomas WHO ° 2 and similar prognosis as benign WHO ° 1 meningiomas
Baumgarten et al. (2016)	141 WHO ° 2 (incl. 20 BIOBM) (Frankfurt series)	Rev.4th, 2016 (neuropathological re-evaluation)	22	No data on degree of resection	BIOBM showed longer PFS vs. atypical meningiomas WHO ° 2
Biczok et al. (2019)	142 WHO ° 1 28 BIOBM	4th, 2007	66	Longer PFS after GTR vs. STR, *p* = 0.001	Shorter PFS of BIOBM than WHO ° I (50 vs. 68 months) however difference did not reach significance
Garcia-Segura et al. (2020)	181 WHO ° 2	Rev. 4th, 2016 (neuropathological re-evaluation)	>48	Longer PFS after GTR vs. STR, *p* = 0.001 GTR defined as Simpson grade I and II	BIOBM showed better prognosis than remaining WHO ° 2 Combination of necrosis and CNS invasion identified as strong predictor of meningioma recurrence
Nakasu and Nakasu (2021)	Meta-analysis (8 studies)	3rd, 2000 4th, 2007 rev.4th, 2016	Unknown	Meta-analysis, thus no data on degree of resection	Brain invasion was a significant predictor of PFS only when both low and high-grade meningiomas have been considered Brain invasion was not prognostic for BIOBM
Banan et al. (2021)	243 WHO ° 1 65 WHO ° 2 (incl. 25 BIOBM)	Rev. 4th, 2016 (neuropathological re-evaluation)	38,2	Degree of resection was not significant for PFS	25 patients with BIOBM showed shorter PFS vs. 243 patients with benign meningiomas WHO grade 1
Behling et al. (2021)	1,412 WHO ° 1 285 WHO ° 2 21 WHO ° 3	3rd, 2000 4th, 2007 (BIOBM outlined as WHO ° 1)	39,6	No data on prognostic role of degree of resection	Positive correlation of CNS invasion and Ki67 proliferation rate
Kim et al. (2022)	Own cohort of 292 meningiomas WHO ° 2 (BIOBM = 7), Meta-analysis (25 studies, 3,590 patients)	3rd, 2000, 4th, 2007 rev.4th, 2016 no central neuropathological review	54 unknown	Longer PFS for own cohort after GTR vs. STR, *p* < 0.001	No consistent association with PFS
Traylor et al. (2023)	339 WHO ° 1 200 WHO ° 2 4 WHO ° 3 (incl. 90 BIOBM)	Rev. 4th, 2016 (neuropathological re-evaluation)	48	Longer PFS after GTR vs. STR, *p* < 0.01	Similar risk of recurrence between BIOBM and WHO grade 1

* The prognostic role of degree of resection refers to the general series and not specific to BIOBM; GTR has been defined as Simpson grade I-III.

The lack of large (prospective) studies with long follow up after complete resections definitely hinders far reaching conclusions regarding the prognostic relevance of brain invasion. However, another possible source of bias, which may contribute to controversial results has been pointed out by Perry, namely the ill-defined criteria for diagnosing brain invasion (Perry, 2021). This may well result in distinctly different rates of CNS invasion reported by different neurosurgical centers (Timme et al., 2020). Indeed, as detailed above the studies reported by Biczok et al., and Banan and co-workers detail a 16.5% vs. 9.3% incidence of BIOBM among otherwise histologically benign meningiomas (Biczok et al., 2019; Banan et al., 2021). While only a slight effect of the classification modification in 2016 on clinical practice had been expected, the increase of cases diagnosed as BIOBM and therefore WHO ° 2 was reported as overwhelming (Timme et al., 2020). Perry described a mini-epidemic of BIOBM in his personal consults, whereas he rejected a sizable number of BIOBM diagnoses during his central review, and discourages neuropathologists from interpretating only focal brain invasion without any additional high-grade features as a criterion for assigning tumors to WHO ° 2. Spreckelsen et al. confirmed Perry’s observation of a large interobserver variability and use of somewhat varying criteria among neuropathologists (Baumgarten et al., 2016). Picart und Spreckelsen et al. point out that precise assessment of CNS invasion is mandatory (Picart et al., 2022; von Spreckelsen et al., 2022). The 5th edition of WHO classification of CNS tumours in 2021 has recognized this issue and suggested more uniformed criteria for the diagnosis of CNS invasion. According to the new classification system, CNS invasion is defined as ‘irregular, tongue-like protrusions of tumour cells into underlying GFAP-positive parenchyma, without intervening leptomeninges. Extension along perivascular Virchow-Robin spaces is not considered to constitute brain invasion because the pia is not breached’ (Timme et al., 2020). Another important aspect of the problem is surgical sampling error (Biczok et al., 2019). Brain invasion may be missed by the neuropathologist because the brain tumor interface has not been or has not been sufficiently sampled during the surgery (Jenkinson et al., 2017; Picart et al., 2022). To this end, Timme et al. reviewed the histological reports of the Neuropathological Institute in Münster, which diagnosed meningioma samples from different Neurosurgical Departments of the region. Since the rate of CNS invasion differed among some neurosurgical departments, he concluded that surgical sampling nuances may impact the accuracy of recognition of CNS invasion (Timme et al., 2020).

## Pathophysiology and molecular profile of CNS invasion

The 2021 WHO classification incorporated for the first-time molecular biomarkers into the diagnosis of grading, like *CDKN2A* homozygous deletion and *TERT* promoter mutation, allowing the assignment of the tumor to WHO/CNS ° 3 even in cases that appear histologically as lower grade (Louis et al., 2021; Table 2). The last edition of WHO classification recognizes also the importance of additional molecular profile analysis, like mutations of *SMARCE1* (clear cell architecture), *KLF4/TRAF7* (secretory meningiomas) and *BAP1* (rhabdoid or papillary morphology) or *H3K27ME3* loss of nuclear expression (potentially adverse prognosis) (Louis et al., 2021). It is now more than obvious, that translational/molecular neuroscience will soon play a key role in diagnosis but also estimation of prognosis and decision making for meningiomas.

**Table 2:** Criteria for histological grade classification of WHO 5th Edition 2021 (Louis et al., 2021).

WHO grade	Description of criteria
Grade 1	Low mitotic rate, <4 per 10 HPFs*
Grade 2	Mitotic rate 4–19 per 10 HPF or Brain invasion** or ≥3 or 5 specific atypical features: • Spontaneous or geographic necrosis, • Patternless sheet-like growth • Prominent nucleoli • High cellularity • Small cells with high n:c ratio or specific morphology: chordoid or clear cell
Grade 3	Mitotic rate > 20 per 10 HPF or specific morphology: papillary or rhabdoid or specific molecular criteria: *TERT* promoter mutation or homozygous deletion of *CDKN2A/B*

CNS invasion has been associated with *AKT1* mutations as well as alterations of metalloproteases and adhesion molecule expression (Jalali et al., 2015; Barresi et al., 2021; Qin et al., 2021). The pathophysiology of CNS invasion seems to undergo different stages (Quintero-Fabian et al., 2019; Maggio et al., 2021; Furtak et al., 2023; Go and Kim, 2023). The crucial point for the initiation of meningioma cells invasion is the cleavage of the basement membrane and the following remodeling of the extracellular matrix (ECM) by specific matrix metalloproteinases (*MMPs*) (Quintero-Fabian et al., 2019; Maggio et al., 2021; Furtak et al., 2023; Go and Kim, 2023). Several activators of *MMPs*, like *uPA* have been linked to plasmin mediated matrix breakdown and cell adhesion (Fleetwood et al., 2014). Kandenwein et al. reported increased levels of plasminogen activator inhibitor-1 *(PAI-1)* in patients with brain invasive meningiomas (Kandenwein et al., 2011). *PAI-1* expression has been identified as a significant prognostic factor (Kandenwein et al., 2011). In a further step, the migration of meningioma cells within the loose environment of the degraded ECM is promoted by adhesion agents, like integrins (Wilisch-Neumann et al., 2013). Finally, well known growth factors, like *EGFR, VEGFR* or *HGF* contribute to neoangiogenesis and growth of the tumor cells (Fleetwood et al., 2014; Go and Kim, 2023). In this regard, Pei et al. reported lower expression of canstatin, an angiogenesis inhibitor, in WHO grade 3 brain invasive meningiomas (Pei et al., 2023). Several other pathways have also been implicated in the biology of meningioma invasion, i.e., *P13K/AKT, FAK, MAPK* and *Hippo* signaling (von Spreckelsen et al., 2022). Alterations in *TERT, BAP1* and *DMD* have been associated with higher histological grade and poorer prognosis (Shankar et al., 2017; Juratli et al., 2018; Samal et al., 2020; Williams et al., 2020; Pellerino et al., 2022).

Roehrkasse et al. (2022) have reported data supporting the concept that the analysis of the molecular background of meningiomas may hold superior prognostic power when compared to histological features. Nassiri et al. (2021) described four consensus molecular meningioma groups with distinct tumour behaviour. Comprehensive molecular profiling of meningiomas should probably include DNA methylation pattern and copy number aberration analyses, investigating mRNA abundance, as well as driver mutations of oncogenes, such as *BAP1, CDKN2A/B*, and the *TERT* promoter (Louis et al., 2021; Nassiri et al., 2021; Roehrkasse et al., 2022). Maas et al. reviewed DNA methylation and copy number aberrations in 3031 meningiomas, and studied mutation data of 858 meningiomas. They merged the molecular and histological data into an integrated molecular-morphological classification score, which predicted more accurately the risk of recurrence than the WHO histological grading alone (Maas et al., 2021).

In summary, these latter studies may indicate that future meningioma grading schemes will increasingly rely on molecular parameters. Nevertheless, the overall number of cases investigated and published is not very large, molecular profiling strategies are complex and time consuming, and vary between investigators. Confirmatory studies are largely lacking. Finally, the quality of the clinic data used for correlations with molecular findings so far is limited, which somewhat precludes drawing robust clinical conclusions already at this point in time.

## Imaging of CNS invasion

**Figure 2:**
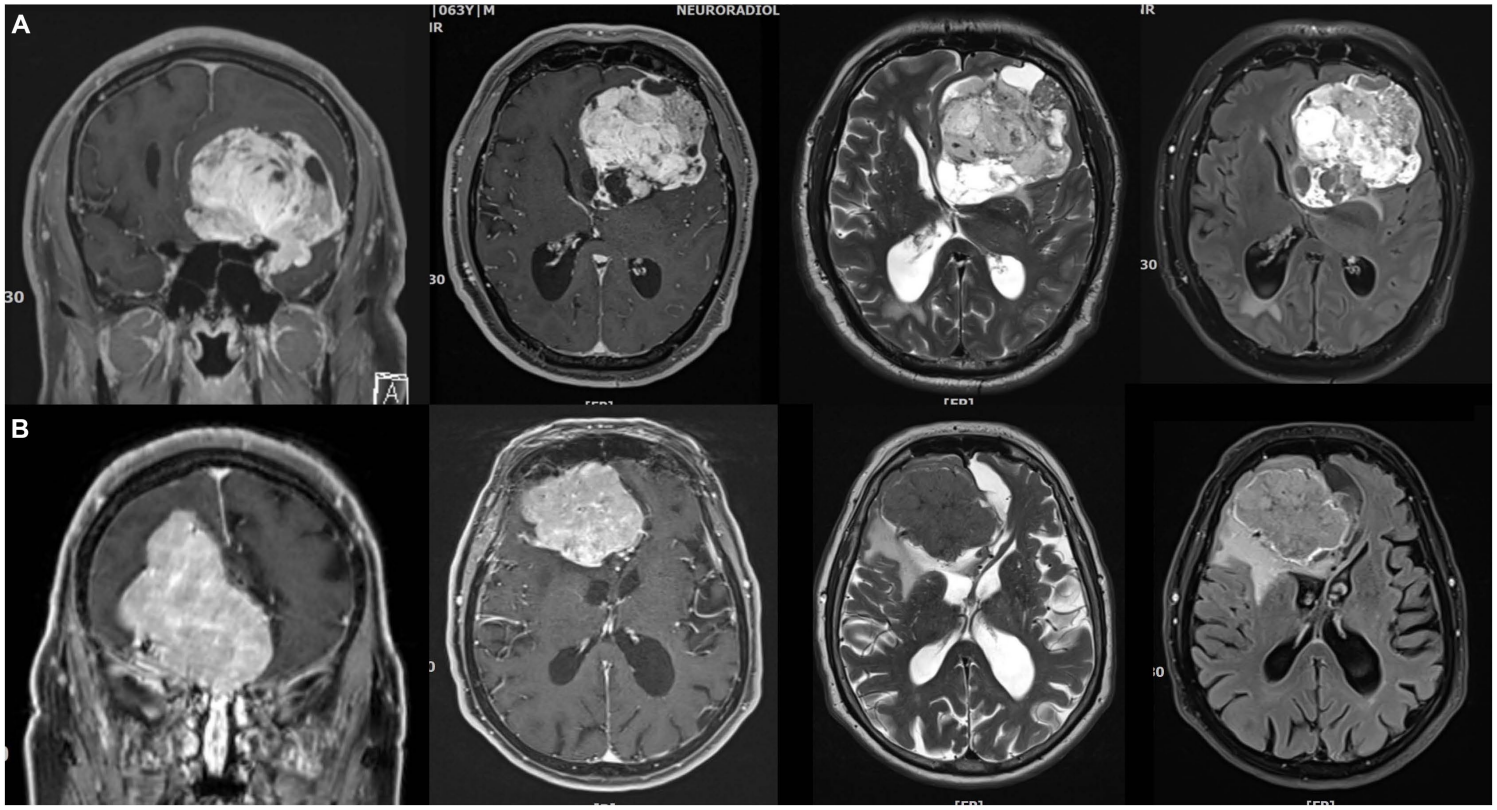
Imperfect correlations between imaging findings, histopathological atypia, and brain invasion (I). **(A)** 64 years old male patient with a very large left anterior clinoidal meningioma assigned to CNS grade 2 based on cytological atypia and an increased mitotic count. However, there was no brain invasion. Somewhat fittingly, MR imaging reveals cysts, a cleft sign and heterogenous contrast enhancement as well as FLAIR and T2 intratumoral heterogeneity, but there was only limited peritumoral edema. **(B)** 82 years old female patient with a large right>left olfactory groove meningioma CNS grade 2. The neuropathological evaluation revealed no atypia, but prominent brain invasion. There is surprisingly little edema. Contrast enhancement is somewhat heterogenous, but the tumor looks rather homogenous on the T2 and FLAIR weighted images.

**Figure 3:**
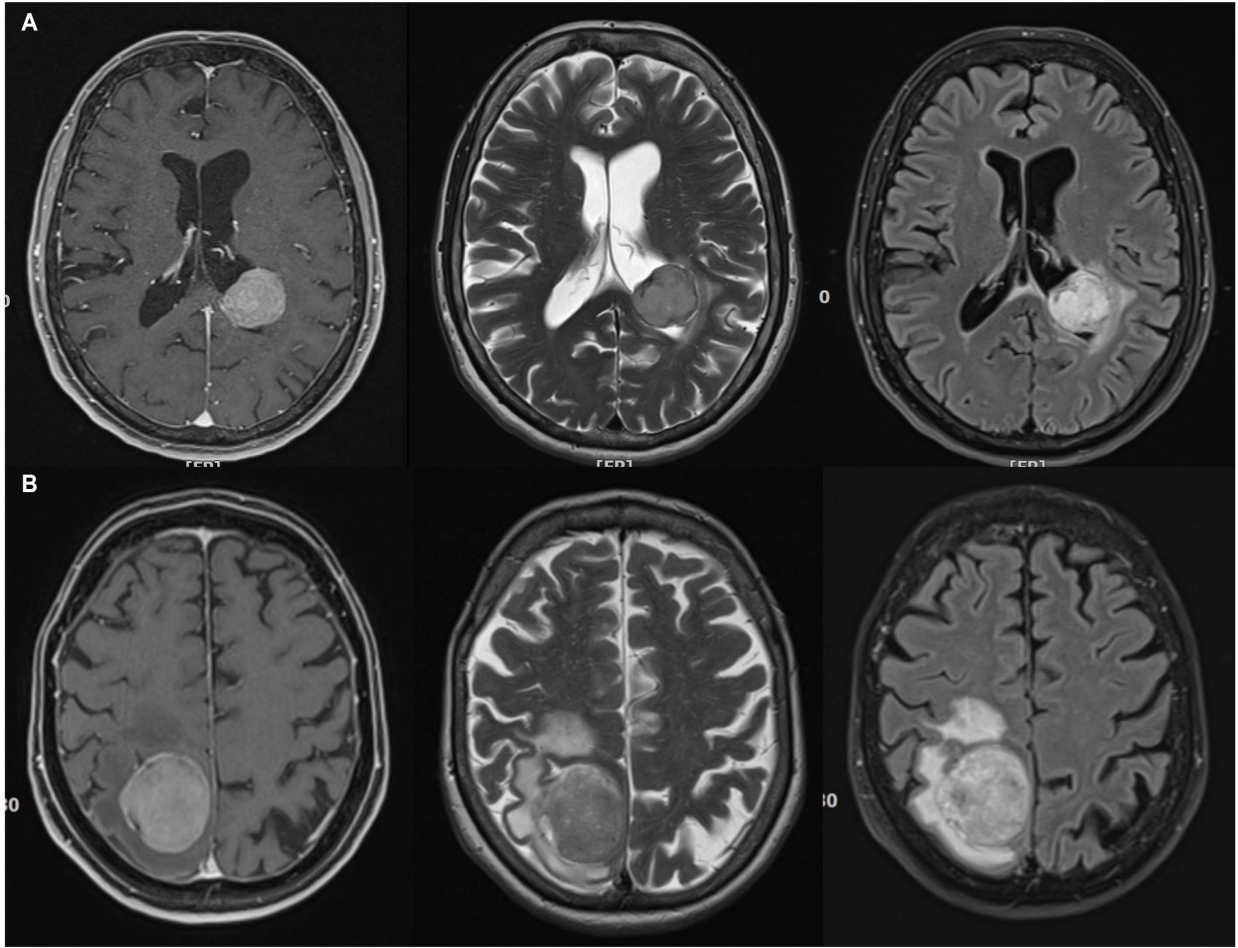
Imperfect correlations between imaging findings, histopathological atypia, and brain invasion (II). **(A)** 74 years old female patient with a left ventricular (trigonal) CNS grade 2 meningioma. This tumor had atypical histopathological features and was found to invade the brain. Possibly in contrast, the MR showed little edema. However, the actual zone of contact between the tumor and the brain parenchyma is very small. T2, FLAIR and contrast-enhanced T1 imaging reveals little heterogeneity. **(B)** 64 years old female patient right parietal parasagittal meningioma CNS grade 2. There was no brain invasion, however, histopathological atypia. Note, that there is substantial edema, while the tumor tissue looks otherwise inconspicuous on T2, FLAIR and contrast-enhanced T1 weighted MR images.

Predicting the grade of meningiomas and brain invasion preoperatively may be advantageous for surgical planning. Basic MR imaging may already help with the identification of brain invasion before the surgery. A higher volume of peritumoral edema as well as heterogeneity regarding tumor morphology and contrast enhancement may suggest an increased risk of brain invasion (Adeli et al., 2018; Joo et al., 2021; Ong et al., 2021). Hyperostosis and bony destruction have been associated with aggressive biological behaviour by some authors (Hanft et al., 2010). The aforementioned signs serve only as ‘warning signs’, though, and are definitely not robust enough to allow for a reliable preoperative diagnosis (Figures 2, 3). Recently, Luo et al. reviewed preoperative MRIs from 543 patients with meningioma WHO grade 1 and 123 with WHO grade 2 including 67 BIOBM and concluded that the imaging features of BIOBM are more similar to WHO grade 2 than 1 (Luo et al., 2023).

Noteworthy, brain invasion as well as meningioma grade may be better predicted prior to surgery by modern high-dimensional quantitative imaging analysis, the so-called radiomics (Zhang et al., 2020; Ugga et al., 2022). Radiomics is increasingly attracting attention in medical oncology, since radiomics-derived nomograms may predict the diagnosis and biological behaviour of different tumours (Lambin et al., 2017). Peng et al. employed radiomics to obtain data from preoperative MRI and cCT studies of 215 patients with benign or high grade meningiomas and established a diagnostic nomogram model for predicting tumor grade based on features like tumor-brain interface, bone invasion and tumor location (Peng et al., 2021). Li et al. (2021) acquired traditional semantic features like tumour volume, location or peritumoral edema as well as radiomic features from the tumour and from the tumour-to-brain interface in a series of 284 meningioma (173 with, 111 without brain invasion), and constructed an integrated nomogram to predict brain invasion. Similarly, Xiao et al. (2021) established a diagnostic nomogram for predicting brain invasion after obtaining radiomic features in 719 patients with meningiomas.

## (Aggressive) surgery for brain invasive meningiomas?

### Oncological benefit from aggressive meningioma surgery

Simpson identified already in 1957 an aggressive meningioma resection as a beneficial prognostic factor (Simpson, 1957). However, nowadays many neurosurgeons recommend more conservative surgeries under the premise that modern adjuvant therapies and imaging follow-up may compensate for incomplete resections. Although these arguments are valid, there is still a risk that patients may forego an oncological benefit that is easy to obtain. If we accept that recurrence rates of meningiomas do not differ significantly with the Simpson grade, resecting the tumor’s dural attachment or bone infiltrations will no longer be rational. Leaving behind tumor tissue in a case with a benign growth may have no adverse midterm consequences, however this may be very different during long-term follow-up (Pettersson-Segerlind et al., 2011). Of note, clinical studies in patients with meningiomas commonly often report only limited follow up, i.e., less than 5 years.

E.g. Sughrue et al. questioned the relevance of Simpson grading of resection in modern neurosurgery, since he indeed found no significant difference in PFS between 373 patients following a Simpson Grade I, II, III, or IV resection for benign meningiomas. However, median follow up was only 3.7 years (Sughrue et al., 2010). A more recent and larger retrospective study on 1,571 patients with meningiomas WHO grade 1,2 or 3 concluded that Simpson grade IV resection was an unfavorable prognostic factor. PFS did not differ between patients with a Simpson grade I vs. grade II resection. Again, mean follow up was only 38 months (Behling et al., 2021). On the other hand, Brokinkel et al. studied 939 patients, who underwent surgery for meningioma of all WHO grades. Median follow-up was 37 months. They found a strong correlation between the Simpson grading and recurrence in general and importantly also between cases with a Simpson grades I vs. II resection. Dichotomizing extent of resection (e.g., gross total vs. subtotal resection) resulted in loss of predictive value (Brokinkel et al., 2021). We have retrospectively analysed 901 patients with meningiomas WHO grade 1 to 3. Median follow-up was 62 months. The estimated 10 years PFS was 91.8 and 81.2% after Simpson grade I and II resections, respectively (Gousias et al., 2016). Thus, coagulation instead of resection of the dural attachment more than doubled the recurrence rate at 10 years in our series. Some groups conducted retrospective cohort studies with a longer median follow up ranging from 85 to 123 months and found a prognostic relevance of the Simpson grades of resection, too (Alvernia et al., 2011; Hasseleid et al., 2012; Winther and Torp, 2017).

It should be noted, that the association between extent or resection (i.e., the Simpson grade) and recurrence seems to be much stronger in tumors with higher WHO grades. Simonetti et al. (2021) investigated 183 higher grade (i.e., WHO grades 2 and 3) meningiomas and found a 5-year survival rate of 95 and 67% after complete or partial resections, respectively. In our study we were able to analyze separately 172 patients with WHO ° 2 tumors. Estimated 10 years recurrence rates were 16% after a Simpson grade I and 50% after a Simpson grade II resection (Gousias et al., 2016). Masalha et al. analyzed retrospectively a cohort of 36 patients with anaplastic WHO ° 3 meningiomas. A complete resection was associated with significantly longer PFS and OS (Masalha et al., 2019). Depei et al. retrieved data for 530 patients from the Surveillance Epidemiology and End Results database who had surgery between 2000 and 2015 and identified a prognostic relevance of a complete resection, in terms of longer PFS, for both cases with WHO ° 2 and 3 tumors (Li et al., 2019).

Since the Simpson grading of resection reflects the subjective intraoperative impression of the surgeon, external imaging-based validation is probably useful. Ueberschaer et al. (2021) validated prospectively the documented Simpson grading through postoperative MRI and 68Ga-DOTATATE/PET-CT and found in 40.5% of the cases unexpected tumour remnants. Along the same lines, Haslund-Vinding et al. (2022) proposed a new (the Copenhagen) grading system for the extent of resection of meningiomas based on a postoperative 68Ga-DOTATOC PET/MRI.

The Simpson grading may not properly account for tumor location (Voss et al., 2017). Schwartz and McDermott have recently reviewed the role of the Simpson grading and suggested to ‘abandon the scale of Simpson grading of resection but preserve the message’ (Schwartz and McDermott, 2020).

### Quality of life and functional outcome after aggressive surgery

Although meningiomas do not always cause neurological deficits or other symptoms, patients with meningiomas demonstrate significant impaired quality of life compared to normative healthy controls even before surgery (van Nieuwenhuizen et al., 2013; Haider et al., 2021). This may be partially attributed to disease-related stress, when a patient realizes that he or she has got a brain tumour, or to preoperative anxiety (Wagner et al., 2019; Haider et al., 2021). Jakola et al. (2012) prospectively evaluated a cohort of 54 patients with meningiomas and found an improvement of the cases’ health related quality of life (HRQOL) after surgery, which was mainly due to relief from anxiety. Miao et al. (2010) reported an improvement of the HRQOL score after treatment, which was nevertheless still worse than the baseline score of healthy controls in a larger cohort of 147 meningiomas. Neurocognitive scores tend to worsen after treatment (Constanthin et al., 2021). A large prospective cross-sectional study of 291 patients with meningiomas WHO ° 1 found a ‘clinically meaningful’ impairment in cognitive functioning after surgery (Nassiri et al., 2019). Sekely et al. reported neurocognitive impairments in 68% of 61 patients treated for a meningioma (surgery, radiation or both). 48% of the patients faced difficulties returning to work (Sekely et al., 2022). Unfortunately, the aforementioned studies have not specifically investigated the potential impact of the degree of resection or brain invasion upon HRQOL.

Methods of assessing of quality of life and neurocognition may differ between researchers and some degree of standardization is probably warranted (Gondar et al., 2021). Functional outcome are easier to study, e.g., in terms of new neurological deficits or performance status scales such as the Karnofsky index. Skull base location, larger tumour volume, but also invasive growth have been associated with and increased risk for postoperative deficits (Ehresman et al., 2019; Maschke et al., 2019; Przybylowski et al., 2020; Haider et al., 2021; Starnoni et al., 2021). The role of the degree of resection has been controversially discussed. Ehresman et al. report a Simpson grade IV rather than complete resection as a predictor of postoperative deficits in a series of 761 patients with meningioma (Ehresman et al., 2019). We similarly found a correlation between adverse Karnofsky outcomes and increasing Simpson grade (Gousias et al., 2016). It is likely that these findings largely reflect incomplete surgeries for more difficult to resect tumours. Along those lines, Schneider et al. described an increased risk for postoperative neurological deficits in patients undergoing radical resections in anterior and posterior skull fossa (Schneider et al., 2019, 2021). However, it is probably also fair to state that more aggressive surgery is not necessarily and always associated with worse functional outcomes (Gousias et al., 2016).

### Surgery of meningiomas with CNS invasion

Only a small proportion of invasive meningiomas are characterized as BIOBM, while the vast majority of tumors with CNS invasion demonstrate additional features of malignancy, like atypia, necrosis and high proliferative capacity (Perry, 2021). As detailed above only few studies investigate specifically BIOBM, and these papers focus on the prognostic value of CNS invasion rather than surgical issues. In lieu of better data, surgical management strategies for these tumors and invasive meningiomas in general should therefore probably reflect the concept of maximal safe resection as well as the relatively strong correlation between extent of resection and recurrence in higher grade meningiomas.

The surgical management of brain invasive meningiomas may pose specific challenges. E.g. Brokinkel et al. (2018) have reported an increased risk of postoperative hemorrhage after surgery for brain invasive meningiomas. However, resection of an infiltrative brain tumor is nothing new for neurosurgeons. The experience gained during glioma surgery could be applied also to surgical cases with brain invasive meningiomas, even if the patterns of invasion are not comparable. Most cases of BIOBM or atypical meningiomas demonstrate slight invasion of pia and superficial cortex, whereas excessive brain parenchyma invasion may be apparent in malignant meningiomas (Perry et al., 1999).

The use of IONM has been reported by several authors. Paldor et al. reviewed forty cases with meningiomas in eloquent areas, mainly adjacent to the sulcus centralis and concluded that IONM may guide the surgical technique and extent of resection in favor of a better postoperative functional outcome (Paldor et al., 2022). Policicchio et al. managed infiltrative lesions of the sulcus centralis, among others also anaplastic meningioma, by IONM but also 3D Ultrasound to identify the tumor-tissue interface. Awake craniotomies may also be helpful. Kumar et al. found awake surgery useful for resections of supratentorial meningiomas during pregnancy (Kumar et al., 2020). Awake craniotomies for meningioma resection may not only maximize the safety of the resection but also result in earlier patient recovery, a reduced length of the hospital stay, ands well as costs (Bakhshi et al., 2021). Shinoura et al. routinely use awake surgery not only for meningiomas compressing cranial nerves (Shinoura et al., 2019) but also in cases with perilolandic tumors and describe a beneficial effect of this technique in terms of less postoperative deficits (Shinoura et al., 2013).

Chakravarthi et al. (2021) routinely incorporate 3D tractography during surgery of anterior skull base meningiomas. Tractography has been used not only in skull base meningiomas, but also in eloquently located meningiomas (Kumar et al., 2014; Zhao et al., 2015). Zhao et al. (2015) reported gross total resection of 11 meningiomas located in the atrium of the lateral ventricle. Surgical planning included tractography. Kumar et al. (2014) confirmed the relevance of tractography use in the surgery of eloquent cortical lesions, among others also in meningiomas.

A more precise intraoperative visualization of tumor margins may also maximize the resections of invasive meningiomas. Advanced optical imaging techniques such as confocal microscopy, optical coherence tomography, and Raman spectroscopy have been used for “optical biopsies,” i.e., intraoperative identification of tumor tissue (Shin et al., 2019). Reichert et al. report an increased glycolytic activity of meningiomas as a possible explanation for their extremely high autofluorescence capacities during a modern visualization technique, namely the flavin mononucleotide fluorescence (Reichert et al., 2023). Charalampaki et al. have recently described confocal laser endomicroscopy which combined with multispectral fluorescence microscopy as a novel technique for intraoperative tumor visualization. The authors report that their technique allows for the depiction of the cellular architecture of tumor margins with 400–1,000 fold magnification (Charalampaki et al., 2019). The ability of confocal microscopy in general to identify brain invasion of aggressive meningiomas has been reported in a mouse model (Peyre et al., 2013). Raman spectroscopy has been used for intraoperative differentiation between meningioma and healthy dura mater (Jelke et al., 2021). Fluorescence-guided microsurgery may also prove helpful when dealing with brain invasive meningiomas (Linsler et al., 2019; Jelke et al., 2021; Chotai and Schwartz, 2022). In order to further assess the benefit of 5-ALA fluorescence-guided meningioma surgery, the NXDC-MEN-301 phase 3 open-label single arm study is currently being conducted in 16 centers of USA, Germany and Austria (Stummer et al., 2022).

## Conclusion

For this paper we have reviewed the more recent literature on meningiomas with histological CNS invasion. From a prognostic point of view brain invasive tumors with additional histological feature of atypia or malignancy are atypical or malignant meningiomas. The prognostic impact of brain invasion as a stand-alone criterion for the diagnosis of an aggressive tumor, however, is not clear. More investigations including larger cohorts of BIOBM will be key for answering this question. The histological analysis of CNS invasion remains the diagnostic gold standard, and more uniform and robust criteria as well as surgical sampling protocols are warranted especially in cases in which only a questionable local brain invasion is suspected (Perry, 2021). It is however not impossible that advanced neuroimaging and high-dimensional image analysis such as radiomics will eventually predict CNS invasion preoperatively (Li et al., 2021; Xiao et al., 2021). Specific molecular markers and correlates for brain invasion are lacking while on the other hand there is considerable progress toward a molecular tumor grading of meningiomas in general.

In lieu of better evidence surgical management of brain invasive meningiomas should follow the principles of a safe, but maximal resection. The extent of resection remains a major predictor of tumor recurrence, and this relation is much stronger in higher grade when compared to benign meningiomas (and by inference therefore quite likely also in brain invasive meningiomas). More conservative surgical attitudes may even be questionable in cases with completely benign tumors since most pertinent studies suffer from limited follow-up, while some nevertheless still provide evidence in favor of radical resections.

Technical adjuncts and techniques which are routinely used in glioma surgery such as intraoperative monitoring, awake craniotomy, DTI tractography, fluorescence-guided microsurgery and ultrasound may help to increase the safety of meningioma surgeries in general and of operations for brain invasive tumors in particular.

## Author contributions

KG: conceptualization. KG and MS: methodology, writing—review and editing, and supervision. KG, LT, and MS: data curation and writing—original draft preparation. All authors have read and agreed to the published version of the manuscript and agreed to be accountable for the content of the work.

## Conflict of interest

The authors declare that the research was conducted in the absence of any commercial or financial relationships that could be construed as a potential conflict of interest.

## Publisher’s note

All claims expressed in this article are solely those of the authors and do not necessarily represent those of their affiliated organizations, or those of the publisher, the editors and the reviewers. Any product that may be evaluated in this article, or claim that may be made by its manufacturer, is not guaranteed or endorsed by the publisher.
